# Socioeconomic health disparities revisited: coping flexibility enhances health-related quality of life for individuals low in socioeconomic status

**DOI:** 10.1186/s12955-016-0410-1

**Published:** 2016-01-12

**Authors:** Saloni Atal, Cecilia Cheng

**Affiliations:** Department of Psychology, University of Cambridge, Cambridge, UK; Department of Psychology, University of Hong Kong, Pokfulam, Hong Kong

**Keywords:** Coping, Flexibility, Health, Quality of life, Socioeconomic status, Stress

## Abstract

**Background:**

Previous research has generally indicated that disadvantaged socioeconomic groups tend to experience poor health-related quality of life (HRQoL). In an effort to extend the literature, this study proposes that coping flexibility is a stress buffer that mitigates the adverse effects of low socioeconomic status (SES).

**Methods:**

The participants comprised 150 Indians (53 % women; mean age = 36.38 years) from high, medium and low socioeconomic groups. Their levels of perceived stress, coping flexibility, subjective SES and HRQoL were assessed individually through household interviews.

**Results:**

The findings provide support for the hypothesised moderating role of coping flexibility between subjective SES and HRQoL (*p* < 0.001). In the low SES group, participants higher in coping flexibility reported significantly better HRQoL than those lower in coping flexibility. Moreover, coping flexibility moderated the association between perceived stress and HRQoL (*p* = 0.001). Of the participants who experienced higher levels of stress, those higher in coping flexibility reported better HRQoL than those lower in coping flexibility.

**Conclusions:**

This study enriches the literature by revealing the beneficial role of coping flexibility on HRQoL among individuals low in SES. These new findings highlight the potential importance of psychological interventions that strengthen the flexible coping skills of socioeconomically disadvantaged groups.

## Background

Socioeconomic disparities in health-related quality of life (HRQoL) are a pressing issue in many countries, especially developing countries with huge wealth gaps [1]. Stress has been identified as one of the primary pathways through which low socioeconomic status (SES) influences HRQoL [2]. However, some emerging studies have shown that not every individual with a low SES level experiences poor health [3–5]. Hence, the ability to cope effectively with stress may be a psychological resource that is particularly relevant to low SES individuals with scant socioeconomic resources [6]. Although some coping studies have examined the effect of SES on health [7, 8], the interplay of SES, coping and HRQoL is not well understood. The present study seeks to address this important but unexplored issue by examining whether coping flexibility mitigates the adverse effects of low SES on HRQoL in India, a developing country with a steep inequality in wealth distribution.

Coping refers to the thoughts and/or behaviour used to manage the demands of a stressful event [9]. These efforts typically aim at directly managing the stressor (i.e., primary approach coping), altering the individual’s stress-related thoughts and behaviour (i.e., secondary approach coping) or stress avoidance (i.e., avoidant coping) [10]. The transactional theory of coping postulates that different stressors have distinct demands, and thus coping flexibility is adaptive [11]. An individual with high coping flexibility tends to adopt primary approach strategies (e.g., problem solving) to handle controllable stressors and secondary approach strategies (e.g., relaxation) to handle uncontrollable stressors [12].

Although a growing body of studies have indicated that flexible coping fosters well-being [13, 14], other studies have similarly shown the adaptiveness of an (inflexible) active coping style [15, 16] We propose that an active coping style may be more beneficial for individuals with a high SES because they are endowed with abundant psychosocial resources, whereas a flexible coping style may be more beneficial for those with a low SES because they have deficient resources. Our proposition stems from the social stress theory, which postulates that individuals lower in SES face numerous and largely uncontrollable stressful experiences but lack the external resources necessary to handle their heightened stress levels [17]. Therefore, an intrapsychic approach that emphasises adjusting one’s self and flexibility may be particularly adaptive for these individuals in terms of stress management [18].

Our proposition is also supported by some resource-based theories of stress. For instance, the reserve capacity model states that individuals lower in SES experience a disproportionate disease burden, partly because psychosocial resources are less accessible to them [19]. When present in abundance, such resources may promote resilience and alleviate resource depletion. Resilience studies have indicated that trauma survivors with initially higher levels of psychosocial resources report better health outcomes over time [20, 21]. Given its association with well-being in highly stressful situations [22], coping flexibility may foster resilience in low SES individuals who experience heightened stress levels.

In summary, the present study aims to test two theory-driven hypotheses. First, the *inner-resource hypothesis* puts forward that coping flexibility may moderate the positive association between subjective SES and HRQoL. Within the low SES group, individuals who display higher levels of coping flexibility may report a better HRQoL than others who display less flexibility. Although we use objective SES to classify participants, we also include subjective SES as a major study variable, as previous studies have indicated that it reliably influences psychological processes and health independently of objective SES [23, 24]. Second, the *stress-buffering hypothesis* proposes that coping flexibility is more beneficial to individuals who experience higher (vs. lower) levels of life stress. Of the individuals experiencing high stress levels, those who are more flexible in coping may experience a higher HRQoL than their counterparts who are less flexible in coping.

## Methods

### Participants and procedures

The participants in this study comprised 150 Indians (71 men, 79 women) who were either born or had spent more than half of their lives in India. Their ages ranged from 18 to 60 years (mean = 36.38; *SD* = 11.99). The participants were initially recruited from a residential community network in Mumbai and then through word of mouth. All of the participants took part voluntarily and none of them received any monetary compensation. Table 1 shows the SES characteristics of the three participant groups, each of which comprised 50 participants. Questionnaires were individually administered through household interviews. The study protocol was reviewed and approved by the university’s research ethics committee. All of the participants signed informed consent forms before the interviews began.

**Table 1:** SES characteristics of study sample

	*Low SES*	*Medium SES*	*High SES*
Variable	*%*	*%*	*%*
Education level			
No school/primary school	76	0	0
High school	24	26	0
College	0	70	20
Master’s level or higher	0	4	80
Monthly household income (Indian Rupees)			
< Rs. 10,000	60	0	0
Rs.10,000 – 30,000	40	24	0
Rs. 30,001 – 50,000	0	76	0
> Rs. 50,000	0	0	100
Occupational status			
Blue collar/service	90	0	0
Clerical	10	52	0
Managerial/professional	0	22	28
Business owner	0	0	36
Other (student, homemaker)	0	26	36

### Measures

#### Coping flexibility

The Coping Flexibility Interview Schedule [25] was used to assess the participants’ coping flexibility. The participants were first asked to describe both the controllable and uncontrollable stressful events they experienced recently along with the strategies they deployed to handle each event. For each reported strategy, they indicated whether the primary goal was to directly manage the stressor (i.e., primary approach coping), change the thoughts and emotions they experienced as elicited by the stressor (i.e., secondary approach coping) or try not to do anything nor think about the stressor (i.e., avoidant coping). A score of 1 was given by a trained coder when a participant deployed primary approach coping in a controllable stressful event or secondary approach coping in an uncontrollable event. A score of 0 was given otherwise. Given that the participants varied in terms of the numbers of stressful event reported, the aggregated scores were divided by the total number of reported stressors for each participant. Thus, the coping flexibility scores ranged from 0 to 1. The Coping Flexibility Interview Schedule displayed good reliability, criterion-related validity and discriminant validity [26, 27].

#### Perceived stress

The Distress Thermometer was used to measure the participants’ current stress levels. This is a visual analogue scale, with scores ranging from 0 (no distress) to 10 (extreme distress) [28]. The section related to health concerns was irrelevant to the present sample and was thus removed. The participants were asked to rate their current stress levels in three domains: work, health and interpersonal relations. The scores across all three of the domains were averaged to provide an estimate of the participants’ current perceived stress levels. A higher score indicated a higher perceived stress level. The Distress Thermometer was found to be both reliable and valid [29].

#### Subjective SES

The MacArthur Scale of Subjective Social Status [30] was used to assess subjective SES. The participants were given a drawing of a ladder with 10 rungs that represented where people stood in Indian society and were instructed to mark the rung that they thought best represented where they stood on the social ladder. A higher score indicated a higher perceived status in society. This scale exhibited good test-retest reliability in addition to discriminant and convergent validity [31, 32].

#### Health-related quality of life

The eight-item Short Form Health Survey (SF-8) was used to assess HRQoL. A summary score was calculated by weighting each of the eight items using a norm-based scoring method [33]. Scores over and under 50 were above and below the average of the general population, respectively. Thus, a higher score reflected a better HRQoL. The SF-8 exhibited high test-retest reliability and discriminant validity [34, 35].

### Data analysis

By way of preliminary analysis, MANOVA was conducted to identify gender and SES group differences in the study variables. Hierarchical multiple regression analyses were then performed to test the hypothesised moderating effects of coping flexibility. In the first step, the predictor variable (subjective SES or perceived stress) and moderator (coping flexibility) were entered into the regression model. In the next step, the interaction term (Subjective SES x Coping Flexibility or Perceived Stress x Coping Flexibility) was added. All of these variables were centred to address any multicollinearity problems. If a significant interaction effect was found, analysis of the simple main effects would be conducted to examine the direction and differences at each level.

## Results

### Gender and SES group differences in the study variables

The MANOVA test revealed no significant gender differences in any of the study variables [Hottelings *T* (4, 144) = 0.047, *p* = 0.22]. In terms of objective SES, there were significant differences in HRQoL, coping flexibility and subjective SES among the three groups (*p*’s < 0.01; see Table 2).

**Table 2:** Objective SES differences in study variables

	Low SES	Medium SES	High SES	*p*
	*N* = 50	*N* = 50	*N* = 50	
	*M (SD)*	*M (SD)*	*M (SD)*	
Subjective SES	3.32 (1.30)_a_	4.92 (0.75)_b_	7.18 (1.34)_c_	<0.01
Perceived stress	5.03 (1.73)_a_	4.90 (1.69)_a_	4.42 (1.64)_a_	0.16
Coping flexibility	0.53 (0.46)_a_	0.19 (0.30)_b_	0.25 (0.38)_b_	<0.01
Health-related Quality of life	59.32 (11.24)_a_	60.30 (8.52)_a_	68.68 (6.96)_b_	<0.01

*Note*: Means in the same row that do not share a common subscript are statistically different by the post-hoc Tukey’s test, *p* < .05

### Moderating role of coping flexibility on the subjective SES-HRQoL relationship

**Fig. 1:**
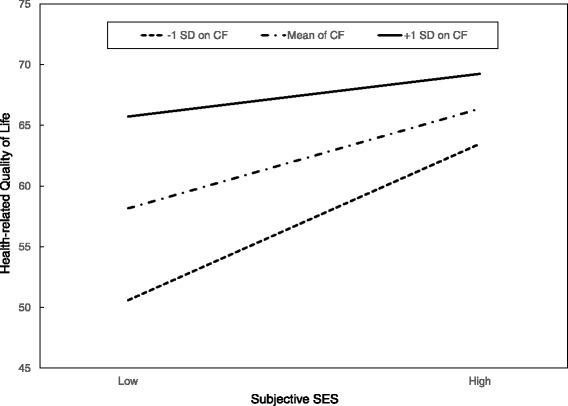
Moderation effects of coping flexibility (CF) on the relationship between subjective socioeconomic status (SES) and health-related quality of life (HRQoL). This graph shows that for participants having low subjective SES scores, those who displayed higher levels of coping flexibility reported significantly better HRQoL life than those who displayed lower coping flexibility (*p* < 0.001). There were no such differences for participants having high subjective SES scores (*p* = 0.55)

As depicted in Table 3, there was a significant interaction effect between subjective SES and coping flexibility [*F* (1146) = 13.99, *p* < 0.001]. This interaction accounted for an additional 5.5 % of the variance in HRQoL after the main effects of subjective SES and coping flexibility had been controlled for. To interpret this significant interaction effect, high and low values for coping flexibility were calculated by adding and subtracting one standard deviation from the sample mean. Analysis of the simple main effects showed that participants in the low coping flexibility-high subjective SES group reported higher levels of HRQoL than those in the low coping flexibility-low subjective SES group (*p* < 0.001). However, there were no significant differences in the levels of HRQoL between the high coping flexibility-high subjective SES group and the high coping flexibility-low subjective SES group (*p* = 0.34; see Fig. 1).

**Table 3:** Multiple regression analyses predicting health-related quality of life from coping flexibility, subjective SES and perceived stress

		Health-related quality of life			
		B	*p*	95 % confidence interval	
				Lower	Upper
Model 1					
Step 1	Subjective SES	2.64	<0.01	1.97	3.31
	CF	11.16	<0.01	7.92	14.34
Step 2	Subjective SES x CF	-2.90	<0.01	-4.43	-1.37
Model 2					
Step 1	Perceived stress	-1.61	<0.01	-2.50	-0.72
	CF	9.86	<0.01	6.16	13.56
Step 2	Perceived stress x CF	-2.90	<0.01	-4.64	-1.11

*Note:* B *= unstandardised regression coefficient*

### Moderating role of coping flexibility on the perceived stress-HRQoL relationship

**Fig. 2:**
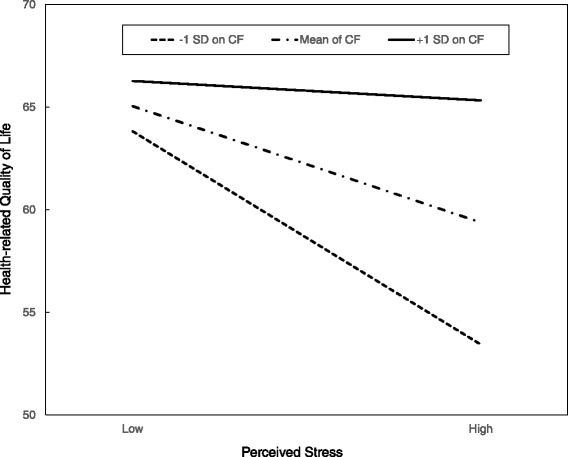
Moderation effects of coping flexibility (CF) on the relationship between perceived stress and HRQoL. This graph shows that for participants who exhibited less coping flexibility, those who perceived higher stress levels reported significantly lower HRQoL than others who perceived less stress levels (*p* < 0.001). No such differences in HRQoL were revealed for participants who displayed higher levels of coping flexibility (*p* = 0.43)

Table 3 also shows that the Perceived Stress x Coping Flexibility interaction was significant [*F* (1146) = 11.12, *p* = 0.001]. The interaction explained an additional 5.8 % of the variance in HRQoL after controlling for the main effects of perceived stress and coping flexibility. Analysis of the simple main effects revealed that the participants in the low coping flexibility-low stress group reported higher HRQoL scores than those in the low coping flexibility-high stress group (*p* < 0.001). However, the high coping flexibility-high stress group and the high coping flexibility-low stress group did not differ in HRQoL scores (*p* = 0.95; see Fig. 2).

## Discussion

This study examines the hypothesised beneficial role of coping flexibility on socioeconomic inequalities in terms of HRQoL. Three key new findings emerge. First, compared with those higher in the SES strata, individuals with a lower subjective SES tend to be more flexible and vary their use of coping strategies according to the perceived controllability of stressful situations. Coping studies have shown that individuals differ in how rigid or flexible they are when deploying coping strategies [36, 37]. Our study is important in that it shows that such individual differences in coping flexibility may be related to one’s socioeconomic environment. This finding can be explained by social class theory of agency [38], according to which individuals have different models of agency that pertain to their normative beliefs about action. Individuals from higher SES strata are endowed with ample socioeconomic resources, resulting in a greater sense of personal control and a tendency to use primary approach strategies to directly tackle problems during stressful encounters. In contrast, individuals with a low SES place value on acceptance and adjustment and thus may tend to be more flexible when deploying different strategies to handle diverse stressors with varying extents of controllability [39] These individuals’ scant resources prohibit them from addressing every problem using primary approach strategies, and an inflexible deployment of primary approach strategies may elicit resource depletion for these individuals.

Second, coping flexibility moderates the link between subjective SES and HRQoL. This result is consistent with those of previous studies, which have documented that psychological factors can act as a buffer against the adverse health effects of low SES. For example, positive reappraisal, a sense of purpose in life and perceived control are associated with better HRQoL in individuals from low SES backgrounds [3, 40, 41]. Individuals from this socioeconomic group are typically faced with frequent and uncontrollable stressors. Therefore, coping flexibility, which entails adapting oneself to the demands of stressful situations, is a highly useful psychological resource for low SES individuals.

Third, coping flexibility moderates the adverse effects of perceived stress on HRQoL. Dual process theory of coping postulates that flexible coping may be particularly useful in highly stressful situations, which often present individuals with varied and fluctuating demands [42]. Studies have identified SES and stress as risk factors that compromise HRQoL [43, 44]. The present study extends the literature by specifying that these risk factors are relevant only to individuals with a lower rather than higher level of coping flexibility. It is particularly noteworthy that among the participants who had higher coping flexibility, the group with low SES and high stress levels and the group with high SES and low stress levels tended to report comparable levels of HRQoL. Such novel results provide converging evidence for the beneficial role of coping flexibility for individuals from the low SES stratum.

The observed stress-buffering function of coping flexibility may have practical implications for the design of intervention programmes. Cognitive-behavioural stress management programmes traditionally focus on building specific skills such as relaxation and problem solving. However, individuals may not be able to apply these skills in a situation-appropriate manner. Thus, stress management programmes should also incorporate a ‘meta-skill’ of coping flexibility that involves teaching individuals how to differentiate stressful situations and deploy coping strategies that best match the specific demands of a situation. For example, a person low in SES may deploy more secondary approach coping in some situations to reserve resources for deploying primary approach coping to handle controllable stressors [45, 46]. Such coping flexibility fosters adaptation to the changing environment for individuals with deficient socioeconomic resources.

Before concluding, some limitations of the study should be noted. First, this study adopted a cross-sectional design and thus the direction of its effect is uncertain. Although coping flexibly may influence HRQoL, it is equally possible that the reverse also holds. Experimental or intervention designs are needed to establish causality. Second, although we observe socioeconomic differences in coping flexibility, it remains uncertain whether these differences are stable over time. Longitudinal studies may be needed to address this issue. Third, the present findings indicate that coping flexibility is a fit between participants’ reported strategies and perceived controllability over stressful events. However, it remains unclear whether the participants perceived that controllability would concur with other people’s judgement. Future studies should include third-party ratings of controllability. Finally, as all of the participants in the study were Indians, its findings should be generalised to other ethnic groups with caution, particularly as there may be cultural differences in areas of stress appraisal and context sensitivity [47, 48]. Notwithstanding these limitations, the present study provides some new evidence that socioeconomic differences in HRQoL may be moderated by psychological resource factors such as coping flexibility.

## Conclusions

In summary, our findings suggest that coping flexibility may be a more crucial correlate of HRQoL than subjective SES and perceived stress. Coping flexibility may be particularly useful to low SES individuals as a psychological resource that can supplement material coping resources and help them to effectively mitigate stress. Furthermore, given that broader social policies and environments are often resistant or take a long time to change, an approach that focuses on enhancing psychological qualities such as coping flexibility may be an effective target point for decreasing socioeconomic health disparities. Future studies should continue to examine socioeconomic influences on coping in general and the construct of coping flexibility in particular.

## Abbreviations

HRQoL: health-related quality of life
SES: socioeconomic status
SF-8: the short form health survey
